# Supplementary material for: ProteinShader: illustrative rendering of macromolecules
Source: BMC Struct Biol. 2009 Mar 30;9:19. doi: 10.1186/1472-6807-9-19 (PMC2672931; doi:10.1186/1472-6807-9-19)
Supplement: Additional file 1 — ProteinShader program without source code. This compressed file contains the complete ProteinShader program including associated libraries, but no source code. A README.txt file gives an overview of the ProteinShader distribution, and the index.html file in the help subdirectory has directions on getting started with the program as well as a set of tutorials. [file 1472-6807-9-19-S1.zip › ProteinShader-beta-0_9_4-binary/help/api/org/proteinshader/gui/ProteinShader.html]

ProteinShader (ProteinShader API)


|  |  |  |
| --- | --- | --- |
| **Overview** \| **Package** \| **Class** \| **Use** \| **Tree** \| **Deprecated** \| **Index** \| **Help** | |  |
| **PREV CLASS**   **NEXT CLASS** | **FRAMES**    **NO FRAMES**     **All Classes** |  |
| SUMMARY: NESTED \| FIELD \| CONSTR \| METHOD | DETAIL: FIELD \| CONSTR \| METHOD |  |


---


## org.proteinshader.gui Class ProteinShader

```
java.lang.Object
  org.proteinshader.gui.ProteinShader
```

---

``` public class ProteinShader extends Object ```

Opens the main GUI window for the ProteinShader program.

---

**Constructor Summary**

`ProteinShader()`


| **Method Summary** | |
| --- | --- |
| `static void` | `main(String[] args)`             Creates an instance of the ProteinShaderGUI and sets it visible. |
| `static void` | `openControlPanel()`             Opens the retractable control panel on the right side of the ProteinShader GUI. |

**Methods inherited from class java.lang.Object**

`clone, equals, finalize, getClass, hashCode, notify, notifyAll, toString, wait, wait, wait`

**Constructor Detail**

### ProteinShader

```
public ProteinShader()
```


**Method Detail**

### main

```
public static void main(String[] args)
```

:   Creates an instance of the ProteinShaderGUI and sets it visible.

    :   **Parameters:**: `args` - command line args are not used by this program.

---


### openControlPanel

```
public static void openControlPanel()
```

:   Opens the retractable control panel on the right side of the ProteinShader
    GUI. If the GUI has not been created yet, this method has no effect (it
    will not crash the program with a null-pointer exception).


---


|  |  |  |
| --- | --- | --- |
| **Overview** \| **Package** \| **Class** \| **Use** \| **Tree** \| **Deprecated** \| **Index** \| **Help** | |  |
| **PREV CLASS**   **NEXT CLASS** | **FRAMES**    **NO FRAMES**     **All Classes** |  |
| SUMMARY: NESTED \| FIELD \| CONSTR \| METHOD | DETAIL: FIELD \| CONSTR \| METHOD |  |


---

# *Copyright © 2007-2008*
